# Synopsis of Discussions, Missouri State Dental Society

**Published:** 1866-04

**Authors:** A. M. Leslie


### Proceedings of Societies.

---

### SYNOPSIS OF DISCUSSIONS, MISSOURI STATE DENTAL SOCIETY.

*At its first Meeting, held Oct.* 31, *and Nov.* 1*st and* 2*d,* 1865.

Reported by A. M. Leslie, by request of the Society.

Dr. I. Forbes suggested, as a method of profitably using the spare time during the organization of the society, that the leading points in practice be taken up, and each member be called upon, by the chair, to state *his mode* of doing the particular thing under discussion, and the *why* he does it so. By such a course the experience of all will be grooped, and each can improve his own practice, by the adoption of so much as he may judge to be better, in that of others.

This proposition being approved, in answer to the question:

### WHY IS DENTAL RUBBER, WHEN VULCANIZED THE SAME TIME AND WITH THE SAME AMOUNT OF HEAT, SOMETIMES HARD AND AT OTHERS SOFT?

Dr. Peebles said, I cannot say what the cause is. The past two years, has used sandarach varnish on his models. Before that, used shellac. He finds that very fine plaster is very hard to cut away after vulcanizing. He prefers coarse plaster in the flask, as he finds it crystalizes, and comes out in a softer state. He does not think pressure necessary to vulcanizing, as he vulcanized a piece on a piece of paper, in a stove.

Dr. Jones thinks over-heating the flask and models, in packing, is the cause of difference in the hardness of different specimens. The rubber, he thinks, is frequently over-heated in packing. Another source is the use of a leaky vulcanizer. The sulphur necessary to the process being in part carried off by the escaping steam. In vulcanizing two pieces, in such a vulcanizer, at one time, he finds the lower one hardest, showing that the lower one gets more heat. This is true to a less extent in a perfect vulcanizer.

Dr. Blake thinks the cause is the difference of dry heat, as applied to the flask in packing.

Dr. Payne attributes the difference to the over-heating of the flasks in packing; uses shellac only as a varnish; adds more heat if steam leaks.

Dr. Cornelius thinks wax, absorbed by model, does not cause any change.

Dr. Spalding considers leaking of steam a cause. A cast brass vulcanizer will not vulcanize, because the steam escapes through the pores. Wildman says shellac spoils rubber.

Dr. Morse thinks leaking of steam the cause.

Dr. Spyer does not consider leaking to be the cause, as he had, when in Brazil, to use a vulcanizer for some time, which would not retain steam, yet he vulcanized; though he had to give it five hours.

Dr. Townsley mentioned a case in which his brother used shellac varnish on his model; this case came out pliable. Before and since, he had no trouble.

Dr. Hovey attributes hardness at some points, and porousness at others, to over-heating flask in packing; also to escape of steam. Formerly used shellac; for two years has used soap; finds this makes a clean piece of work.

Dr. Sloan holds, that rubber may be subjected to the same amount of heat in packing as in vulcanizing. The difference originates in the use of dry heat in packing, also from escaping steam. Hurrying at a high heat, makes it porous, especially if thick. If vulcanizer leaks, keep up the heat by adding more heat under the vulcanizer. Vulcanizes at 330° for forty-five minutes. In using thermometer first time, notes carefully the point the mercury reaches when the vulcanizing is right, and files a notch there, and is guided by that in preference to the scale.

Dr. Hale considers the difficulty originates in too high heat in packing. Has seen mercury and sulphur on the surface of pieces, showing an imperfect mixing or, decomposition of these materials. Porousness, he considers, is caused by rapid heat. He vulcanizes forty-five minutes at 320°.

Dr. Payne holds that thick pieces must have much longer time, at a lower degree of heat. Had tested a half inch piece beside a thin piece, in ordinary time, and found the thin good and the other spoiled. Vulcanized a piece one inch thick, six hours, at 270°; it came out good. He vulcanized some pieces in half an hour, at 340 to 350. In lower thick pieces, uses slips of platina embedded in the rubber; these form centres, which prevent burning. One case which he vulcanized thirty minutes, at 363, was worn six months, when patient complained of a metalic taste when he eat fruits or acids. He vulcanized it thirty minutes more; this removed the cause of complaint.

Dr. McKellops exhibited some specimens of English rubber work. One for regulating with, possessed some good points.

Dr. Clark understands that pressure and heat are necessary to vulcanizing.

Dr. Morse attributes the separation of the rubber from some teeth, to the presence of wax and oil; avoids this by boiling the flask in water before packing.

Dr. Leslie stated a case where he vulcanized with the safety plug blown out of a Hayes' vulcanizer.

Dr. McCoy considers the fact of plaster being a slow conductor of heat, we frequently do not make due allowance for this. The thermometer generally feeling the influence of the heat, sometimes in advance of the rubber.

### HOW DO YOU DESTROY THE TOOTH PULP?

Dr. Clark said: Is very careful to remove all foreign and decayed matter from the surface of the investing membrane or pulp. Applies a very *minute* portion of arsenic, and covers with loose cotton, as he deems it a cardinal principle, in treating a pulp, that where there is no pressure there is no pain:

therefore has abandoned the use of arsenic, made up in the form of pill, with other substances; also the use of varnishes in the cotton covering.

Dr. I. Forbes, in applying arsenic, dips a pledget of cotton into sandarach varnish; takes up a small portion of arsenic and creosote on the point of this pledget, and inserts it in the cavity, after cleansing the same.

Dr. Hovey uses a paste made of arsenic and morphine; applies a small portion on cotton, covers loosely with another piece saturated in sandarach varnish. In straght fangs, has the patient generally return in twenty-four hours; in other cases, allows thirty hours. In some cases, thinks he has seen the action of arsenic extend through the dentine, for teeth when extracted have been colored. In cases of lower molar, the covering of *wax* had escaped, and the arsenic produced ptyalism.

Dr. McCuen prepares the cavity by fully exposing the nerve; he makes a concave cap for the nerve, of wood; under this he places the arsenic, and seals the cavity with wax. In approximal cavities, places a wedge gently between the teeth, to insure retention of arsenic, &c.

Dr. McCoy cannot add anything to Dr. Forbes' mode, which he procured of him two years ago and still pursues.

Dr. Peebles uses one part crystalized arsenious acid and four parts tannin, made into a paste, with creosote. Cannot always thoroughly expose the pulp. Takes three hatchet-shaped excavators; on one he puts a pledget of drying cotton, on one cotton and a small portion of the above preparation; on the other, raw cotton and varnish; avoids pressure. Applies from twenty-four hours to three weeks.

Dr. Sloan uses, chemically, pure arsenic. Exposes the nerve; carefully applies a small portion of arsenic, on cotton; covers with cotton and a strong solution of collodion; leave in five hours.

Dr. Prevost exposes nerve as much as possible; uses one part morphine, one part arsenic, closes lightly with wax; time, twenty-four hours. Thinks nerve cannot be killed without pain, unless fully exposed.

Dr. Payne, in very sensitive cases, applies creosote alone, for twenty-four hours; can then remove the dead bone easily; uses morphine and creosote to destroy pulp; plugs with a ball of wax, using heat to soften it; never plugs with cotton, as it absorbs moisture. Applies about twelve hours.

Dr. Morse has used arsenic and morphine, and arsenic alone; never saw any good from use of morphine; uses cotton mixed with wax over the application; time, twenty-four hours; had a stubborn case recently, when made three applications; then applied equal parts of alcohol and creosote, with as much tannin as they would dissolve.

Dr. Comstock removes decay: moistens a small piece of cotton with creosote; plugs with different substances.

Dr. Reed uses a paste of arsenic and morphine; covers with cotton; time, six to twelve hours; has not found advantage in the use of wax.

Dr. Goodrich treats like others; has had some bad results; attributes it to too much arsenic; in one case he had to remove a portion of the alveolus, from its bad effects, and names his as a caution to others.

Dr. Blake removes decay carefully; uses a paste of morphine, arsenic and creosote; plugs with sandarach and cotton, taking great care to avoid pressure.

Dr. McKee uses arsenic paste, applies to nerve, and plugs with wax.

Dr. Clark uses arsenious acid directly to nerve, and seals with creosote. Is opposed to the use of tannin, because it cuts off absorption; opposes the use of morphine, because arsenic is the best narcotic; covers with a piece of soft cotton. If the arsenic is preserved from the saliva, a half hour is enough to suffice.

Dr. Judd does not fear the constitutional effect of arsenic; generally uses a twentieth of a grain, which is a safe dose; the length of time is unimportant, in a white soft tooth, two hours is sufficient; in a dense tooth, twenty-four will do no harm; generally removes in two or three hours.

### INFLAMMATION OF PERIOSTEUM AND ITS TREATMENT.

Dr. Judd said: the subject covers the whole ground of medicine. Inflammation *per se* is the most discussed subject in medicine. One theory of inflammation succeeds another. One says it arises from stagnation points, that is, at some points some portion of the blood will settle. Under a powerful glass, it is observed that the red corpuscles of the blood occupy the centre, and the white creep along the sides. When inflammation sets up, a derangement takes place. Two are seen to become attached, these to a third; some become attached to the sides, and so on; soon clogging up the capillaries and arresting circulation. These accumulations or attachments are broken up or separated in two ways; one is by gradual separation, the other by destruction of the cells.

For subduing inflammation of the dental periosteum, he relies upon the use of seline cathartics and lancing.

Dr. Clark has never been able to subdue inflammation between the fangs.

### ABSORPTION OF THE ALVEOLAR PROCESSES, CAUSED BY THE PRESENCE OF TARTAR.

Dr. Judd does not think it yet established that this absorption is caused by inflammation. Sometimes absorption is seen when there is no tartar on the teeth. It is yet a question whether the tartar acts by pressure on the alveolus or on the gum. The absorption produced by pressure in regulating teeth, may aid us in arriving at correct views. Has not, as yet, read anything which satisfied him how absorption is caused when there is no tartar.

(TO BE CONTINUED.)

---

INFANT MORTALITY.—Of every hundred newly-born children, according to English life insurance tables, Dr. Wm. Farr says, twenty-six die in the first five years.

